# Editorial: Insights in healthcare professions education: 2024

**DOI:** 10.3389/fmed.2025.1760309

**Published:** 2026-01-13

**Authors:** Jacqueline G. Bloomfield, Lynn Valerie Monrouxe

**Affiliations:** The University of Sydney, Darlington, NSW, Australia

**Keywords:** health profession education, innovation, methodology, pedagogy, technology

## Introduction

We are pleased to present the 2024 edition of the *Insights in Healthcare Professions Education* Research Topic. This Research Topic of 14 articles highlights innovations in educational design, research, and evaluation methods shaping healthcare professions education. Four themes emerge: 1. Pedagogical Innovation, 2. Non-traditional Teaching Models, 3. Technology Integration, and 4. Curriculum Design, Accreditation, and Global Collaboration. The Research Topic also features two literature scoping reviews. We hope these articles offer valuable insight into how educators and researchers worldwide are addressing the challenges of preparing future healthcare professionals with the skills and knowledge needed to deliver safe, compassionate, person-centered healthcare.

## Pedagogical innovation

The authors of three articles in this theme recognize the evolving landscape of contemporary healthcare and propose innovations in pedagogy.

Jerjes and Harding advocate for a paradigm shift in medical education through the integration of patient feedback into clinical training. Their article critiques the traditionally clinician-centric model and argues for a more inclusive, patient-centered approach. They propose that embedding patient perspectives fosters empathetic, socially conscious practitioners and ensures curricula are aligned with real-world healthcare needs. The authors highlight how patient feedback can enrich student learning, strengthen communication skills, and enhance treatment outcomes. Ultimately, the article positions patient feedback not only as a pedagogical tool but also as a catalyst for cultural change in healthcare, advancing inclusivity, responsiveness, and improved patient outcomes.

In their thought-provoking commentary, Dong et al. examine how psychological safety can be fostered across health professions education. Drawing on five years of global workshops, they apply Clark's four-stage model to diverse contexts, each with challenges such as hierarchy, performance pressure, or technology. The article outlines strategies to address these threats and stresses cultural sensitivity, noting that perceptions of safety differ across settings. The authors advocate embedding psychological safety into curricula, faculty development, and institutional policies, particularly to advance diversity, equity, and inclusion.

Mohiyeddini introduces Self-Directed Teaching (SDT), a framework supporting educator autonomy and growth. Rooted in theories of self-directed learning, self-determination, and constructivism, SDT promotes reflection, goal setting, resource use, and ongoing evaluation. It helps educators manage higher education's complexity, adapt to diverse student needs, integrate technology, and reduce burnout. SDT is presented as proactive and flexible, emphasizing autonomy and intrinsic motivation amid institutional pressures. Though innovative and theoretically informed, it remains conceptual, lacking empirical evidence. Future research is needed to assess its application. Nonetheless, the article reframes educator development as a dynamic, self-guided process and offers a foundation for further exploration

## Non-traditional teaching models

Evaluating the effectiveness, acceptability, and feasibility of non-traditional teaching models remains a central focus in health professions education, with three articles grouped in this theme examining new approaches for healthcare professionals and students in China.

Luo et al. evaluated a blended learning approach based on the ADDIE (Analysis, design, Development, Implementation, Evaluation) model in training newly hired nurses. Involving 87 participants, the study compared traditional training with a structured program integrating online modules, simulations, and interactive strategies. Results indicated that the blended learning group outperformed the control in knowledge, skills, self-directed learning, and critical thinking, with higher scores in motivation, planning, self-management, and communication. Teaching satisfaction was also greater. The study highlights the value of learner-centered, technology-enhanced education and structured instructional design in nursing.

Shen examined the BOPPPS (Bridge-in, Objective, Pre-assessment, Participatory learning, Post- assessment, and summary) teaching model in ward rounds for newly graduated nurses. Unlike teacher-centered methods, BOPPPS emphasizes engagement through structured phases. The study of 260 students compared BOPPPS-based and traditional groups. Results showed the BOPPPS group achieved higher scores in knowledge, skills, and clinical competence, with greater satisfaction, stronger communication, and improved critical thinking. Teachers noted better performance but increased workload. The study highlights BOPPPS's effectiveness in fostering technical and soft skills, while stressing the need for institutional support. Broader adoption and further research into long-term impact are recommended.

Zong et al. surveyed 923 healthcare staff to assess palliative care training. Fewer than half had received training, which was linked to improved knowledge, confidence, and attitudes. Offline lectures were common but less effective than case-based and online video learning, while community projects proved ineffective. Pain management and communication remained weak, with misconceptions persisting. The study highlights the need for behavior-oriented programs tailored to professional roles and calls for stronger palliative care education in emergency settings.

## Technology integration

Technology is increasingly shaping healthcare education and was the was the focus of three articles within the Research Topic.

Salman et al. evaluated the performance of three generative AI tools (ChatGPT-4, Microsoft Copilot, and Google Gemini) on 75 cardiovascular pharmacology questions. ChatGPT showed the highest accuracy across formats and difficulty, Copilot performed well in short answers but declined in advanced multiple-choice, while Gemini lagged, especially in complex scenarios. The study highlights ChatGPT and Copilot's strengths for foundational and intermediate learning, cautions against reliance for advanced reasoning, and notes the importance of prompt design and free-access limitations. It concludes that AI can enhance healthcare education but requires careful integration, with further research needed on broader subjects and model refinement.

Suzuki et al. evaluated high-fidelity 3D-printed sinus models as alternatives to cadavers in endoscopic sinus surgery (ESS) training. Seventeen otolaryngologists performed identical procedures on both, assessed with OSATS and time metrics. Results showed no significant differences, with strong correlations across measures. The study suggests 3D models provide comparable training value while offering reproducibility, standardization, and accessibility. Unlike cadavers, they can be mass-produced, customized, and used without ethical or logistical barriers. The authors advocate broader adoption of simulation-based training, particularly where cadaver access is limited, and call for further validation in the clinical setting.

Sałacińska et al. in Poland examined medical students' perceptions of two high-fidelity simulation formats: traditional manikin-based scenarios and virtual patient simulations. Results showed no significant differences in problem-solving, teamwork, engagement, satisfaction, or confidence. Female students rated active learning more positively in the virtual format, while older and advanced students reported greater satisfaction overall. The study suggests both approaches are equally valuable for clinical and interpersonal skill development, with demographics influencing preferences, and supports including both modalities in medical training for adaptable learning.

Collectively, these articles stress technology as serving pedagogy, not replacing it. AI aids foundational knowledge but struggles with complex reasoning (Salman et al.). Virtual and traditional simulation perform similarly (Sałacińska et al.), while 3D models expand training options and address ethical and access issues (Suzuki et al.). Technology is most effective when tackling challenges such as accessibility, ethics, and standardization, rather than replacing mentorship, judgement, empathy, or professional identity in healthcare education.

## Curriculum design, accreditation and global collaboration

Three studies included in this Research Topic highlight how aligning healthcare education with local needs, engaging stakeholders in curriculum design, and fostering interdisciplinary or international collaboration can drive meaningful and sustainable improvements in education, training and practice.

Almaghaslah examined pharmacy program accreditation and performance on the Saudi Pharmacists Licensure Examination (SPLE) using data from 27 colleges and over 7,500 students. Accreditation, national or international, was linked to higher pass rates, with ETEC-accredited colleges showing significantly better outcomes. Government-funded institutions outperformed private ones, and top universities held only national accreditation, suggesting ETEC alone ensures readiness and compliance. The study questions the added value of international accreditation for established public institutions and calls for further research into student-level factors affecting SPLE outcomes.

Amid growing calls for sustainability in healthcare, Wieërs et al. examined integrating the UN Sustainable Development Goals (SDGs) into medical, pharmacy, and biomedical education. Surveys at the University of Namur showed strong support, though many educators were unsure how to apply specific goals. The authors propose linking SDGs to medical topics and advocate systems thinking and interdisciplinary collaboration to address barriers such as time, training, and silos. Students emphasized that SDGs must be embedded through active pedagogy and institutional coherence, not treated as an add-on.

Acheampong et al. presents a co-creation model between universities in Ghana and Canada to strengthen primary health care. Through the Africa Health Collaborative, five short courses were delivered to over 100 providers, involving joint planning, content development, and stakeholder engagement. Results showed high satisfaction, increased knowledge, and demand for further training. The study highlights the value of equitable partnerships in global health education and potential replication in other low- and middle-income countries.

## Reviews

Two further studies included in this research topic sit outside these themes but take the form of scoping reviews.

Matschl et al. reviewed 126 studies on ultrasound education in obstetrics and gynecology, finding wide variation in curricula, formats, and assessments. Ultrasound's operator-dependent nature requires structured training, with evidence supporting early undergraduate integration. Effective strategies include peer teaching, dyad learning, simulation, and e-learning, though cost and access remain barriers. Standardized tools exist but need refinement, and the authors advocate multimodal, competency-based curricula with institutional standardization.

Yousuf et al. examined 24 studies on breaking bad news in the Eastern Mediterranean. Professionals showed willingness to disclose but lacked training, awareness, and protocol adherence, with cultural factors shaping practice. The review calls for culturally sensitive education and qualitative research to improve communication in diverse healthcare settings.

## Discussion: cross-cutting themes and synergies

While the articles in this Research Topic address diverse topics across healthcare professions education, several powerful cross-cutting themes can be identified when examined collectively, revealing interconnections across educational innovations.

A central theme across these contributions is the shift from teacher-centered transmission to learner-centered transformation. Models such as ADDIE (Luo et al.), BOPPPS (Shen), autonomy frameworks (Mohiyeddini), patient feedback (Jerjes and Harding), and psychologically safe environments (Dong et al.) illustrate learners and educators as active agents. Grounded in constructivism and self-determination theory, this transformation requires autonomy, competence, relatedness, and psychological safety.

Traditional assessment is challenged by a vision of multifaceted evaluation—technical metrics, expert observation, simulator data, peer review, AI verification, and patient feedback. OSCEs capture proficiency, patient perspectives reveal empathy, expert review assesses reasoning, and teamwork emerges through observation (Sałacińska et al.). Technology, including AI (Salman et al.), simulation (Sałacińska et al.), and 3D models (Suzuki et al.), expands options but cannot replace human judgement.

Communication appears as a foundational competency across studies on breaking bad news (Yousuf et al.), psychological safety (Dong et al.), patient feedback (Jerjes and Harding), palliative care (Zong et al.), and technical training (Matschl et al.; Shen). Equity and cultural responsiveness are likewise essential, linked to psychological safety, diverse patient representation, and global frameworks such as the SDGs (Wieërs et al.). Co-creation partnerships (Acheampong et al.) further exemplify equitable collaboration.

Educational innovation requires institutional support including time, development, and teaching communities, to prevent burnout. Psychological safety must extend to educators (Mohiyeddini; Dong et al.), and faculty development must address implementation (Wieërs et al.).

Finally, Navigating the tension between standardization and contextualization reveals “structured flexibility”: frameworks that guide without constraining. Models such as ADDIE (Luo et al.), BOPPPS (Shen), psychological safety (Dong et al.), and co-creation (Acheampong et al.) exemplify this balance. Grounded in complexity theory, effective systems require adaptable frameworks that maintain coherence. National accreditation standards can ensure quality without international standardization when tailored to local contexts (Almaghaslah).

## Conclusion

These articles highlight holistic approaches recognizing practice as synthetic, where complex patient needs demand integrated deployment of knowledge, skills, judgement, communication, and professionalism. Embedding multiple competencies in authentic contexts better prepares professionals than fragmented methods.

As healthcare education evolves, this Research Topic offers insights into reimagined pedagogies and partnerships: learner-centered paradigms prioritizing psychological safety, assessment as multifaceted ecosystems, communication and cultural competence as foundational, technology serving pedagogy without replacing human elements, and institutional support enabling sustainable innovation.

Across diverse countries and professions, these innovations reflect a shared commitment to preparing adaptable, compassionate, globally aware professionals. Excellence requires holistic transformation addressing pedagogy, assessment, equity, technology, and institutional support together.

We hope this Research Topic sparks further innovation and dialogue, encouraging educators and researchers to continue shaping the future of healthcare education with creativity, rigor, and purpose.

